# Profiles of Social-Emotional Readiness for 4-Year-Old Kindergarten

**DOI:** 10.3389/fpsyg.2017.00132

**Published:** 2017-01-31

**Authors:** Michele M. Miller, H. Hill Goldsmith

**Affiliations:** ^1^Department of Psychology, University of Illinois SpringfieldSpringfield, IL, USA; ^2^Department of Psychology, University of Wisconsin–MadisonMadison, WI, USA

**Keywords:** temperament, social-emotional development, preschool, school readiness, child development

## Abstract

Children who are viewed as ready for kindergarten and/or first grade typically exhibit high attention, approach, and adaptability coupled with low activity and reactivity. These characteristics tend to be especially valued by teachers and describe a child who is “teachable,” or school ready. Since many children enter formal schooling earlier by attending pre-K for 4-year olds, often called 4-year-old kindergarten, there is a need to examine school readiness earlier than kindergarten, which may look very different developmentally. If we expect children to enter formal schooling at age 4, then it should be clear what we expect of them in order to succeed. We explored which temperament, behavior, and cognitive items teachers of 4-year-old kindergarten (*N* = 29) rated as highly characteristic versus uncharacteristic of ready 4-year-olds. This teacher-generated data identified five clusters of children who were deemed ready for 4-year-old kindergarten. Teachers noted high cognitive skills and following directions as salient in many of the clusters, which aligns with the readiness expectations for kindergarten and first grade. However, items that distinguished the five clusters from one another referenced differences in activity level, sociability, shyness, enthusiasm, and patience that were not expected based on the previous literature with slightly older children. Given that some of the children teachers identified as especially ready for 4-year-old kindergarten did not fit this static model of a “teachable” child, a single profile of school readiness at an early age may be inappropriate.

## Introduction

Much of the existing literature on school readiness concerns entering 5-year-old kindergarten or first grade. However, in the United States, 5-year-old kindergartens are no longer seen as transitions from the home to formal schooling, as formal school entry often occurs prior to age 5. Instead, in kindergarten children are expected to learn content formerly taught in grades 1 and 2 (Farran, 2011). As the pressure to succeed academically is imposed on children earlier than ever before, there is understandably widespread interest in assuring that children are prepared to enter the classroom environment early and thrive once there. Researchers emphasize the need to consider readiness prior to kindergarten entry, with some even advocating beginning in infancy (Wilen, 2003; Snow, 2006; Lally, 2010). Readiness to start school is a multifaceted construct that encompasses not only cognitive aspects of children’s development but also social-emotional aspects. The component of social-emotional development deserves increased attention in the consideration of a child’s readiness for early education. Temperament theory provides a useful framework for conceptualizing this aspect of development. Differences in temperament effect how children interact with the world around them, learn, and develop. Knowledge of childhood temperament can help identify why some children are more prepared for formal schooling than others. Moreover, a consideration of temperament theory may facilitate educators’ use of classroom management strategies and interventions to provide children with varied temperaments an equal chance at early academic success (Keogh, 2003). Although the majority of children in the United States are prepared for formal schooling (Pianta et al., 2007), kindergarten teachers judge approximately one-third of children as unprepared for kindergarten-level work (Wilen, 2003; Ramey and Ramey, 2004).

### Teachers’ Ideas of School Readiness

While readiness is typically measured using chronological age and mastery of cognitive skills, it also concerns expectations regarding the abilities and skills a child should enter the classroom with to be successful (Espinosa et al., 1997). Skill-focused assessments fail to align completely with teachers’ perceptions of readiness, which is also social and behavioral (Hains et al., 1989). A survey conducted in the United States of America of more than 7,000 kindergarten teachers conducted by the Carnegie Foundation for the Advancement of Teaching (Boyer, 1991) indicated that teachers judged children as unready for the following reasons: problems with language (88%), emotional immaturity (86%), lack of general knowledge (83%), and lack of social confidence (80%). Preschool teachers often encourage talking as it promotes communication and language skills (Hadley et al., 1994) and rank basic social interaction and communication among the most important skills for children to acquire (Hains et al., 1989). Kindergarten teachers generally rate social-emotional and communication skills as more important than cognitive skills such as counting, identifying colors and shapes, knowing the alphabet, and problem solving (Davies and North, 1990; Heaviside, 1993; Lin et al., 2003).

### Temperament and Social-Emotional Readiness for School

Temperament, individual differences in behavioral reactivity and regulation, is directly related to these essential social-emotional and communicative skills (Goldsmith and Harman, 1994). Reactivity is a broad domain of temperament that refers to one’s reaction to internal and external stimuli. Regulation, specifically self-regulation, refers to processes that moderate (either enhance or inhibit) reactivity (Rothbart and Bates, 2006). Differences in temperament affect how children interact with the world around them, learn, and develop. Knowledge of childhood temperament can help identify why some children are more prepared for formal schooling than others.

The temperament dimensions of adaptability and approach are significantly related to school achievement and have high loadings on a “social adaptability” factor (Martin, 1989; Martin et al., 1994). Temperament dimensions of activity level, distractibility, and persistence, which load highly on a “task orientation” factor, are also highly related to being ready for the transition to school. The literature widely recognizes low activity, high persistence, and low distractibility as characteristics of temperament associated with academic success (Martin and Holbrook, 1985; Martin et al., 1988; Martin, 1989; Orth and Martin, 1994). A study of teachers of 5–7 year olds in England revealed that the ideal pupil was low in reactivity, adaptable, and high on the “task orientation” factor (Klein and Ballantine, 1988). The children that teachers classify as “teachable,” or ready, can be differentiated from other students in the classroom in terms of their temperament. A child who is able to regulate activity level and emotionality, focus attention, persist, and withstand distractions is seen as more ready for formal instruction compared to a child who does not have these temperamental tendencies (Keogh, 1986). Children who exhibit this “teachable” profile are especially valued by teachers (Keogh, 1982, 1986; Martin, 1989). However, a recent study finds that children with a low task orientation (high activity and distractibility and low persistence) can lead teachers to increase behavioral control, which improves the students’ academic success (Vilijaranta et al., 2015). Emotional well-being and academic success likely have a bidirectional effect whereby feeling unready for the challenges of schooling can generate an array of negative emotional reactions from the child (Raver, 2002). Children who have difficulty paying attention, following directions, getting along with others, and controlling negative emotions are not as prepared for school (Arnold et al., 1999; McClelland et al., 2000; Raver and Knitzer, 2002). Risk factors in the United States for poor social-emotional well-being and early school readiness include low income, low maternal education, and having a single parent (Ferguson et al., 2001; Raver and Knitzer, 2002; Ramey and Ramey, 2004). Some 32% of kindergartners face one demographic risk, and 16% face two or more (Raver and Knitzer, 2002). Because temperament can influence teaching style and interpersonal interactions with students, educators need to recognize these biases to better support children with various temperaments in the classroom.

A child’s ability to self-regulate is strongly linked to academic learning. Specifically, self-regulation and attention are cited as two main components of learning-related social skills (McClelland and Morrison, 2003). Children who are able to focus and pay attention will perform better academically upon entering school (Alexander et al., 1993). Self-regulation measured with a delay of gratification task at 54 months predicted behavioral and academic school readiness in kindergarten even after accounting for maternal behaviors (Razza and Raymond, 2013). Essential to the concept of self-regulation is the construct of effortful control, defined as “the efficiency of executive attention—including the ability to inhibit a dominant response and/or to activate a subdominant response, to plan, and to detect errors” (Rothbart and Bates, 2006, p. 129). Effortful control involves attention shifting and focusing, as well as inhibiting or activating behavior. Effortful control is associated with high academic performance and this association is mediated by social competence with peers and teachers as well as classroom engagement (Eisenberg et al., 2010; Zhou et al., 2010).

Developing these important regulatory aspects of temperament is essential for both the initial adjustment to school and continued academic success as they provide the foundation for adaptive classroom behavior (McClelland and Morrison, 2003; McClelland et al., 2006). Relevant social skills for adaptation to the classroom context, which are linked to self-regulatory characteristics of temperament, include interacting positively, cooperating, sharing, and respecting others (McClelland et al., 2000). Preschoolers who struggle with emotion regulation and social relationships have difficulty succeeding in school (Raver and Knitzer, 2002), as social-emotional competencies predict later classroom adjustment and kindergarten readiness (Denham et al., 2014).

Temperament differences can help explain why some students are able to develop and maintain positive relationships with their peers and teachers while others cannot. The development of social skills such as social information processing and social competence prior to kindergarten entry is essential for early school readiness (Ziv, 2013; Robinson and Diamond, 2014). Children who do not express themselves in a socially appropriate way are more likely to be victimized by peers, while those who are rated as compliant and less disruptive are more likely to be accepted by their peers following the transition to kindergarten (Goldsmith et al., 2001). Appropriately expressing one’s emotions can lead to positive interactions with others. Positive affect promotes the development of socially appropriate emotional expression. A child who is highly positive is likely to show increased and sustained empathic responding (Robinson et al., 1994). Early positive affect predicts the development of prosocial empathy-related helping behavior in 2-year-olds (Volbrecht et al., 2007). The development of empathy fosters concern and helping behavior toward others, leading to positive social interactions. Children who are prone to negative affectivity (e.g., anger, sadness) may be less likely to forge positive relationships with others. Children who are inactive, withdrawn, and quiet are also at risk for adjustment problems because they tend to approach others less and are especially sensitive to feedback (Balck et al., 1998). Forming quality relationships is essential for adjusting to the classroom, and certain aspects of temperament lend themselves well to positive social interactions, while others do not. Generally, children who are better at self-regulating their emotional states are apt to experience more positive peer relationships (Stocker and Dunn, 1990; Rubin et al., 1995). Troublesome relationships with peers can lead to poor achievement in kindergarten and a negative attitude toward school in general (Ladd, 1990; Welsh et al., 2001; Raver, 2002).

### Gender Differences in Early School Readiness

Girls are slightly more ready for school at an early age than males (Zill, 1999; DiPrete and Jennings, 2008). At age five, girls performed significantly higher on a Peabody Picture vocabulary test of verbal ability than boys, and boys had more problems with attention than girls (Cooper et al., 2011). In another study, girls scored higher than boys on 8 out of 10 preschool self-regulation tasks (Denham et al., 2012). Thus, at least at an early age, girls seem to display key characteristics of school readiness to a greater degree than boys. A comprehensive meta-analysis of gender differences in temperament examined studies with children from the ages of 3 months to 13 years. Girls scored higher on effortful control and lower on activity level than boys (Else-Quest et al., 2006). This female profile is highly consistent with the “task orientation” factor characteristic of school-ready children.

### The Present Study

Here, we explore teachers’ bases for judgment of readiness for a formal pre-K program for 4-year-olds, often referred to as 4-year-old kindergarten.^1^ Using temperamental, behavioral, and cognitive items, teachers of 4-year-old kindergarten identified characteristics of actual children they recalled as being most ready, or prepared, for school in the initial weeks of entering their classrooms. Although the characteristics of young children ready for 4-year-old kindergarten could have been inferred from the literature cited above, fresh data are needed given that extant data are not specific for early readiness. Based on the literature with slightly older children, we predicted that teachers of 4-year-old kindergarten would describe a “teachable” child by selecting temperament and behavior items related to high levels of emotion regulation and persistence, and low levels of activity and distractibility as highly characteristic of a 4-year-old child who was ready for school. Children who don’t fit any of the ready profiles are probably not optimally ready and that is an issue for different research. Presumably, teachers and parents should work toward building skills and attitudes that move children toward at least one of the ready profiles. Not being in a ready profile isn’t a sentence to failure; it just suggests that further adaptive development needs to be supported.

## Materials and Methods

### Participants

A sample of local licensed teachers of 4-year-old kindergarten (*N* = 29), who had completed at least 1 year of teaching, described ready 4 year olds. The sample of teachers of 4-year-old kindergarten identified themselves as exclusively White and female, with one teacher of Latino origin. Teachers ranged in age from 26 to 60 years (*M* = 41.17 years, *SD* = 9.457). Teachers reported having 2–31 years of teaching experience overall (*M* = 12.79, *SD* = 7.93) and 1–24 years of experience specifically teaching 4-year-old kindergarten (*M* = 6.52, *SD* = 5.77).

### Procedure

This study was carried out in accordance with the recommendations of the Institutional Review Board of the University of Wisconsin–Madison with written informed consent from all participants. All participants gave written informed consent in accordance with the Declaration of Helsinki. Administrators and principals at 20 public school districts and six private schools provided permission to contact teachers of 4-year-old kindergarten programs. Thirteen of the districts and three of the private schools agreed to participate, two districts declined (one due to lack of interest and one because it was the first year of their 4-year-old kindergarten program), and we were unable to confirm contact for five districts and three private schools. From these districts and schools, 60 teachers were contacted for this study. Twenty-nine teachers agreed to participate, and we were unable to confirm contact for 31 teachers. No teacher directly declined to participate.

Teachers completed a consent form, demographics questionnaire, and two Q-sorts of cognitive, behavior, and temperament items. The term “Q-sort” identifies a methodology that focuses on ordering attributes within persons; it derives from a factor analytic technique that operates on correlations between subjects across variables (Block, 1961). These sorts identified specific commonalities among the characteristics of children whom teachers had previously taught and believed were especially ready for their classrooms. To ensure the teachers were comfortable with this Q-sort method, a practice sort of 10 items describing a celebrity was performed with guidance prior to the actual readiness sorts. Additionally, the examiner was present during the entire assessment to answer questions. Teachers received a $25 gift card for their participation.

### Items for the Readiness Q-Sorts

We identified and modified items from two sources for the Q-sorts.

#### Questionnaire Measure

##### The Children’s Behavior Questionnaire (CBQ -106 item version, Rothbart et al., 2001)

The CBQ is a parent-report instrument that assesses temperament in a very broad fashion in children ages 3–8 years. To assess children’s readiness for 4-year-old kindergarten, a selection of 60 items from the CBQ activity level, anger, approach, attention focusing, inhibitory control, sadness, shyness, smiling and laughter, and attention shifting scales were used. We selected items for the present analysis based on relevance to the school context and teachers’ ability to evaluate them. Items on the CBQ that were considered too parent-specific (e.g., “Sits quietly in the bath,” “Rarely gets upset when told s/he has to go to bed”) were not included in this analysis.

#### Observational Measure

##### Bayley Scales of Infant Development-Second Edition (Bayley, 1993)

This individually administered examination assesses the current development and functioning of children ages 1–42 months. We used items from the Mental Scale and Behavior Rating Scale (BRS) in this study. We used 9 dichotomously scored items that assess memory, habituation, problem solving, early number concepts, generalization, and classification. The BRS examines qualitative aspects of the child’s test taking behavior. We included these items because children’s behavior in a novel situation with an experimenter may be comparable to their behavior in a new school setting with a teacher, and therefore could be evaluated by the teachers in this study. Twenty-one items were used in this analysis.

### Determination of Ready Clusters by Teachers of 4-Year-Old Kindergarten

Twenty-nine teachers of 4-year-old kindergarten programs performed two separate Q-sorts each to identify the characteristics of two children (one boy and one girl) whom they deemed more prepared for their classrooms than others. Temperament, behavior, and cognitive items from the CBQ, the BRS, and the Bayley Mental Scale, respectively, were used to create the 90 items for the Q-sort (see Supplementary Material for the complete list of items). Again, item selection was based on appropriateness for teacher consideration and relevance to school readiness. Items on the CBQ that we considered too parent-specific (e.g., “Sits quietly in the bath,” “Rarely gets upset when told s/he has to go to bed”) were not included. Items from these measures were converted into statements that reflected the hypothesized positive direction for readiness as described by the literature. Specifically, the set of items reflected high levels of cognitive skill, controlled behavior, orientation/engagement, emotional regulation, skilled gross and fine motor movement, coordination, approach, attention focusing, inhibitory control, and smiling and laughter, and low levels of activity, anger, sadness, shyness, and attention shifting. Each item was printed on its own 3 × 5 index card.

Each teacher was asked to consider one boy and one girl whom she felt was especially ready for their 4-year-old kindergarten classroom. Teachers were told that these two highly ready children could be quite different from or very similar to one another. The Q-sorts required teachers to place each of the 90 index cards into one of nine small sleeves lined up on the table. The first sleeve (numbered “1”) was for cards that were least descriptive of the child, while the ninth sleeve (numbered “9”) was where teachers placed cards that best described the child. Teachers received instructions to begin by making three broad piles (not necessarily containing the same number of items) of “not like this child,” “somewhat like this child,” and “a lot like this child” and then to sort the items into the nine categories. Items “not like this child” were divided into sleeves 1–3, “somewhat like this child” into sleeves 4–6, and “a lot like this child” into sleeves 7–9, with switching between broad initial piles when necessary. The Q-sort technique forced teachers to make multiple comparisons among the items in the sort because each of the nine sleeves needed to contain exactly 10 cards. After each sort was completed, the placement (sleeve 1–9) of each of the 90 items was recorded.

Cluster analysis of the teachers’ sorts identified characteristics of children they considered ready for 4-year-old kindergarten. The final clusters depicted hypothetical 4-year-olds considered ready for school by teachers. These final clusters had an average placement score for each of the 90 items. We expected a maximum of two or three clusters for each gender, some of which might be similar for both girls and boys.

### Statistical Approach

We employed K-means cluster analysis using SPSS on the Q-sort data to reveal common clusters of hypothetical children who were deemed ready for 4-year-old kindergarten across teachers. *Although this approach does not yield statistical criteria for best fit, it allows for an examination of a range of cluster solutions*. Because teachers identified the sex of the children used to perform the Q-sorts, the clusters were also examined for gender differences. Furthermore, we considered the placement of the various assessment item types (i.e., temperament, behavior in a novel testing environment, cognitive) in the sorts.

## Results

### Creation of Different Clusters of Ready Children

Teachers sorted two sets of 90 index cards, once for a specific boy they recalled as being particularly ready for 4-year-old kindergarten, and once for a specific girl. The order of the sorts and cards were randomized to prevent order, or practice effects. Twenty-nine correlations were performed, one for each teacher, comparing each teacher’s placement of the 90 items for the boy versus girl sort. The data set was transformed so that each teacher was a variable and each card item was a case in order to run these correlations. Twenty out of the 29 teachers produced very similar sort results for both the boy and girl chosen. This is evident by the moderate to high significant positive associations between the sorts (range: *r* = 0.30 – 0.78). Five other correlations were moderate and positive, although these failed to reach significance (range: *r* = 0.11 – 0.20). Only three out of the 29 teacher sort correlations were near zero, and only one teacher significantly sorted the boy and girl characteristics differently (*r* = -0.40). Based on this pattern of associations, teachers generally described ready boys and girls similarly.

**FIGURE 1:**
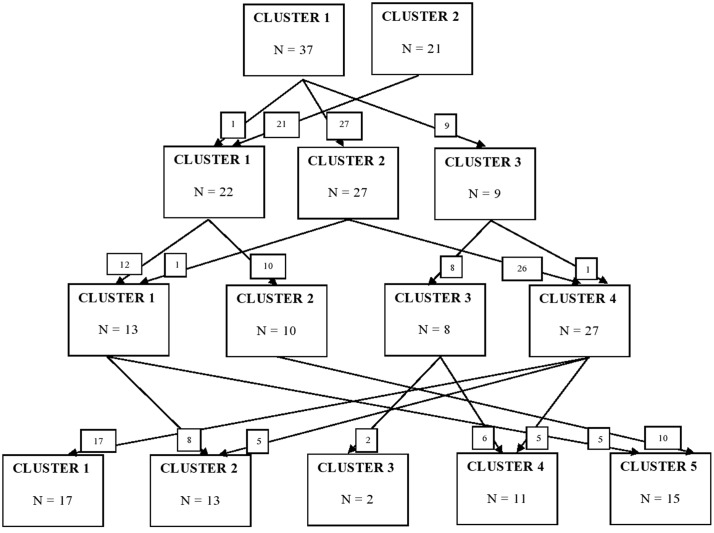
**Movement of children across clusters as more clusters were extracted.** Cluster designations (e.g., Cluster 2) are arbitrary and don’t coincide from solution to solution.

Using K-means cluster analysis, we explored solutions of differing numbers of clusters. **Table 1** provides a description of items that were noted as characteristic (high, sleeves 7–9) versus uncharacteristic (low, sleeves 1–3) of a ready child on each of the clusters for the five cluster solution. **Figure 1** depicts children’s movement across these differing cluster solutions as the number of clusters increased from two to five. In a two cluster solution, both clusters described a child with high cognitive skills. However, the child in cluster 1 was attentive and cooperative while the child in cluster 2 was seen as active and easily frustrated. Moving to a three cluster solution provided these same descriptions from the two cluster solution with an added third that described a child who was attentive, persistent, but not overly enthusiastic. The four cluster solution retained this third cluster from the three cluster solution. Clusters 1, 2, and 4 all described children who had high cognitive skills but were differentiated by characteristics of enthusiasm, high sociability, and shyness, respectively. The addition of a fifth cluster in the 5-cluster solution presented a new, unique profile (cluster 3, *n* = 2) of a child who is rated highly on the characteristics of positivity, enthusiasm, sociability, and cooperation, but not on items of cognitive skill. This 5-cluster solution additionally makes cluster 4 more distinct and homogeneous, as compared with the 4-cluster solution. Three of the clusters in the 5-cluster solution (numbers 1, 2, and 5) are unchanged as far as membership, from the 4-cluster solution. Determining the final number of clusters to form is challenging because no standard, objective selection procedure exists. We ultimately chose a five cluster solution based on one consideration that has been shown to have notable success, using the relative increase in *R*^2^ and the interpretability of the resulting clusters (Grimm and Yarnold, 2000). *R*^2^ represents the amount of variance in the data explained by the cluster groupings, which increases as the number of clusters increases. When the change in *R*^2^ is minimal, additional clusters are not needed to explain the variation in the data. A two cluster solution yielded an *R*^2^ of 0.16, while a three cluster solution increased the *R*^2^ value by 0.06–0.22. From a four to five cluster solution *R*^2^ additionally increased from 0.24 to 0.25, while movement from a five to six cluster solution only increased the value of *R*^2^ by 0.004. Characteristics noted as highly descriptive for many of the clusters were high cognitive skills and following directions. Items that distinguished the clusters from one another pertained to activity level, sociability, shyness, enthusiasm, and patience. Based on the correlations between the boy and girl sorts as well as the representation of both boys and girls within each cluster, the clusters were not strongly differentiated by gender. Therefore, we did not establish the ready clusters separately for boys and girls. No significant group differences were found in the clustering of the items based on teachers’ age, years of experience teaching 4-year-old kindergarten, or years of experience teaching overall based on ANOVA.

**Table 1:** Interpretations of the 5 clusters.

		Characteristic items	Uncharacteristic items
1	5 boys, 12 girls	Identifies pictures, colors, and shapes; Compares sizes; Identifies incomplete pictures; Completes patterns; Classifies; Typically on task; Attentive; Follows instruction	Joins others quickly and comfortably; Not easily mad or frustrated; Smiles at friendly strangers; Likes to sit quietly and watch; Often does not seem to hear me when working
2	9 boys, 4 girls	Identifies pictures, colors, and shapes; Compares sizes; Shows enthusiasm; Cooperates; Follows instruction	Prefers quiet activities; Not easily mad or frustrated; Likes to sit quietly and watch
3	1 boy, 1 girl	Displays heighted positivity, interest, initiative and enthusiasm; Explores objects and/or surroundings; Persistent; Attentive; Sociable; Cooperates; Good gross- and fine-motor skills; Prefers to join other children playing rather than watch; Completes task before moving on; Not mad if mildly criticized; Follows instruction	Identifies incomplete pictures; Not hyperactive; Not embarrassed by attention from strangers; Prefers quiet activities; Concentrates; Not easily frustrated; Usually excited; Smiles a lot at people including strangers; Likes to sit quietly; Not easily angered; Often does not seem to hear me when working
4	3 boys, 8 girls	Identifies colors and shapes; Cooperates; Not hyperactive; Patient; Sits still; Concentrates; Follows instruction; Not easily distracted	Gets very excited; Not embarrassed by attention from strangers; Does not sit quietly outside; Not shy around new people; Often giggles and acts silly; Smiles at friendly strangers; Often does not seem to hear me when working
5	11 boys, 4 girls	Identifies pictures, colors, and shapes; Appropriate gross motor movement; Prefers to join other children playing rather than watch	Not in a big hurry; Patient; Prefers quiet activities; Sits still; Inhibits inappropriate laughing or smiling; Not easily angered; Likes to sit quietly and watch

### Description of the Ready Clusters

As depicted in **Table 1**, cluster one (*N* = 17: 5 boys, 12 girls) identified a child who was cognitively skilled (e.g., knows colors, shapes, patterns), was on task, attended well, followed instructions, and was low on being comfortable with strangers and not becoming angry or frustrated. Cluster two (*N* = 13, 9 boys, 4 girls) identified a child who also had high cognitive skills and followed instruction. This child was also enthusiastic and cooperative. Cluster two was low on preferring quiet activities to active games and not becoming angry or frustrated. The hypothetical child identified on cluster three (*N* = 2, 1 boy, 1 girl) was highly positive, enthusiastic, persistent, inquisitive, social, and cooperative. This child was rated low on non-hyperactivity, preferring quiet games, friendliness toward strangers, and not becoming angry when provoked. Cluster four (*N* = 11, 3 boys, 8 girls) described a child who was rated as highly patient, cooperative, and focused. This child was also cognitively skilled and followed instruction. The child in cluster four was rated low on social items, especially low on social interactions/skills with strangers. Finally, cluster five (*N* = 15, 11 boys, 4 girls) described a child who was highly social and cognitively skilled. This child was rated low on items dealing with patience, low activity (i.e., highly active), and not becoming angry (i.e., did tend to become angry).

## Discussion

### Multiple Pathways of Early School Readiness

As predicted, temperament and behavior differences existed among children selected as especially ready for 4-year-old kindergarten. Although the literature widely recognizes one static “teachable” profile especially equipped for success in school marked by low activity, high persistence, and low distractibility (Keogh, 1982, 1986; Martin and Holbrook, 1985; Martin et al., 1988; Martin, 1989; Orth and Martin, 1994), the teachers in this study identified multiple clusters of children whom they believed were especially prepared for 4-year-old kindergarten. This provides some encouraging evidence that different pathways toward becoming school-ready exist.

Interestingly, the teacher-created readiness clusters for boys and girls were positively associated, suggesting that ready 4-year-old boys and girls share similar characteristics. However, several teachers mentioned that it was much easier to select and describe the female child. This is likely because girls possess the “teachable” temperament characteristics of lower activity level and higher regulation to a greater degree than boys at this age (Zahn-Waxler et al., 2008). Moreover, the clusters did not differentiate by gender. Therefore, the most ready girl and boy seemed to share characteristics related to being identified as school ready. Although the temperament literature suggests that girls are more ready (Keogh, 1982, 1986; Martin, 1989; Else-Quest et al., 2006), characteristics of early school readiness are shared by boys and girls.

These common characteristics of ready children grouped into five clusters. High cognitive skills were present in all but one of the five (cluster 3). Teachers consistently rated high cognitive skills as one of the most characteristic aspects of readiness. It is unsurprising that the majority of teachers identified cognitively skilled children because children who enter school with higher academic skills do achieve at higher levels in elementary school (Duncan et al., 2007).

Along with cognitive skills, many of the clusters identified the ability to follow instruction and cooperate. This finding aligns with research on slightly older children, which indicates that children who attend well, follow directions, and get along with others are better prepared for school (Arnold et al., 1999; McClelland et al., 2000; Raver and Knitzer, 2002). Cooperation is one necessary social skill for successfully adapting to the early classroom context (McClelland et al., 2000).

Clusters were differentiated by children’s degree of sociability, proneness to anger and frustration, activity level, enthusiasm, and patience. Among the children identified as ready, some were more social than others. This observation aligns with the finding from the literature that both inhibited and uninhibited temperament types are susceptible to difficulty with interpersonal relationship formation, a component of school readiness (Kagan and Snidman, 2004; Coplan and Arbeau, 2008). Differences evident in the expression of negative affect, activity level, enthusiasm, and patience were more novel. Under the assumption of a single profile of a “teachable” or ready child, one would expect low negative affect, low activity level, high enthusiasm, and high patience (Keogh, 1982, 1986; Martin, 1989). Some of the children whom teachers identified as especially ready for preschool did not fit this expectation, suggesting that--at least at this early age--a single profile of early school readiness is inappropriate.

### Strengths

We examined early school readiness earlier than the majority of the literature, at age 4. Teachers (the experts) provided a detailed, multifaceted description of ready children at age 4 by defining clusters of ready children using 90 items of temperament, behavior, and cognition.

### Limitations

A shortcoming of this project was that specific children were not followed-up with academic achievement data. Instead, teachers were asked to simply consider children who were especially ready for their 4-year-old classrooms. Moreover, we did not ask teachers to describe children who they deemed unready, which would have provided an interesting comparison. An additional factor compromising the generalizability of the findings was the lack of diversity in the sample of teachers. The exclusively female and White teachers represented the population of teachers of 4-year-old kindergarten programs from local suburban areas. Teachers’ own temperaments might also have been important considerations in how they rated ready children. This study did not measure teachers’ temperaments or personality factors and thus did not consider teacher–student fit. Results could differ substantially upon considering a more varied group of teachers and school contexts.

### Future Directions and Implications

Our results could best be extended by longitudinally following a large sample, with ample diversity in families and teachers, of children from infancy to school entry and beyond. Information on children’s temperament, behavior, and cognition should be obtained at various points in development from their parents and teachers, as well as trained observers. In such an extension, teachers should not only report on children’s academic achievements, but also on their behavior and temperament. Furthermore, experimenters should observe children’s behaviors within the classroom environment in addition to the laboratory setting. Ideally, the sample would be followed extensively enough to analyze the predictive effects of early school readiness on multiple measures of academic success over the years. Our results set the basic expectation that multiple profiles of readiness should be identified in such complex longitudinal studies.

It is especially noteworthy that experts, teachers of 4-year-old kindergarten, defined the characteristics of ready children in this study. Student–teacher interactions are influenced by teachers’ perceptions of children’s school readiness, which directly and indirectly influence children’s success in the classroom. If children are expected to enter formal schooling at the age of 4, whether they are prepared to do so or not, then parents and educators should have realistic expectations for what readiness for this early classroom environment should look like. This will also help to better prepare children to succeed in the classroom once there. Understanding which aspects of temperament and behavior teachers note as most characteristic of early readiness may help caregivers to prepare children to meet the demands of early schooling successfully.

## Author Contributions

Both authors contributed extensively to the work presented in this paper. MM designed the experiment and collected data for this project. HHG supervised the work. Both authors conducted data analysis, discussed the results and implications of the work, and commented on the manuscript at all stages. MM drafted the article and HHG critically revised the entire manuscript. MM and HHG approved the final version for submission.

## Conflict of Interest Statement

The authors declare that the research was conducted in the absence of any commercial or financial relationships that could be construed as a potential conflict of interest.

## Footnotes

1 It is important conceptually for researchers and educators to understand the multiple components of readiness, and we contribute to this understanding. Our examination is not an applied contribution. We are not providing a recipe for estimating readiness in practice, nor do we empirically investigate ways to increase readiness. Whereas practice may be more oriented to overcoming deficiencies, we instead focus on the positive aspects of school readiness.
