# Supplementary material for: Profiles of Social-Emotional Readiness for 4-Year-Old Kindergarten
Source: Front Psychol. 2017 Jan 31;8:132. doi: 10.3389/fpsyg.2017.00132 (PMC5281560; doi:10.3389/fpsyg.2017.00132)
Supplement: Supplementary file 1 [file Data_Sheet_1.docx]

*Appendix*

List of items for the teachers’ Q-sorts (original measure in parentheses):

1. Can correctly identify pictures (of common objects). (Bayley Mental Scale)

2. Can correctly label/name pictures (of common objects). (Bayley Mental Scale)

3. Can correctly identify colors (primary colors). (Bayley Mental Scale)

4. Has knowledge of basic shapes (e.g. circle, square, triangle). (Bayley Mental Scale)

5. Can make comparisons of size. (Bayley Mental Scale)

6. Can correctly label/name colors (primary colors). (Bayley Mental Scale)

7. Can identify incomplete pictures. (Bayley Mental Scale)

8. Can complete basic patterns. (Bayley Mental Scale)

9. Can classify objects. (Bayley Mental Scale)

10. Often displays heighted positive affect. (BRS)

11. Rarely displays negative affect. (BRS)

12. Easily soothed when upset. (BRS)

13. Not hypersensitive to stimuli. (BRS)

14. Typically relinquishes materials and accepts new materials. (BRS)

15. Displays much interest in stimuli. (BRS)

16. Consistently shows initiative with tasks. (BRS)

17. Often explores objects and/or surroundings. (BRS)

18. Typically on task, attends well. (BRS)

19. Typically persistent. (BRS)

20. Typically displays enthusiasm toward tasks. (BRS)

21. Typically trusting, rarely fearful. (BRS)

22. Very rarely frustrated with inability to complete tasks. (BRS)

23. Typically responsive, rarely avoidant or resistant. (BRS)

24. Makes many attempts to interact socially. (BRS)

25. Cooperates well. (BRS)

26. Has appropriate gross-motor movement. (BRS)

27. Has appropriate fine-motor movement. (BRS)

28. Good coordination of movement. (BRS)

29. Typically has appropriate timing and pacing of movement. (BRS)

30. Not hyperactive, not fidgety or agitated in movement. (BRS)

31. Does not always seem to be in a big hurry to get from one place to another. (CBQ)

32. Can lower his/her voice when asked to. (CBQ)

33. Usually prefers to join other children playing, rather than watch. (CBQ)

34. Laughs a lot at jokes and silly happenings. (CBQ)

35. When picking up toys or other jobs, usually keeps at the task until it's done. (CBQ)

36. Rarely gets irritated when s/he makes a mistake. (CBQ)

37. Seems to be at ease with any person. (CBQ)

38. When s/he sees a toy s/he wants, gets very excited about getting it. (CBQ)

39. Tends to walk rather than run from room to room. (CBQ)

40. Does not have temper tantrums when s/he doesn't get what s/he wants. (CBQ)

41. When s/he wants to do something, s/he talks about little else. (CBQ)

42. Does not get embarrassed when strangers pay a lot of attention to him/her. (CBQ)

43. When practicing an activity, keeps his/her mind on it. (CBQ)

44. Does not seem to feel "down" at the end of an exciting day. (CBQ)

45. When outside, does not often sit quietly. (CBQ)

46. Enjoys funny stories and usually laughs at them. (CBQ)

47. Acts very friendly and outgoing with new children. (CBQ)

48. Completes a task before moving to another one. (CBQ)

49. Joins others quickly and comfortably, even when they are strangers. (CBQ)

50. Not frustrated when prevented from doing something s/he wants to do. (CBQ)

51. Does not usually become tearful when tired. (CBQ)

52. When mildly criticized will not get mad. (CBQ)

53. Can wait before entering new activities is s/he is asked to. (CBQ)

54. Does not have difficulty waiting in line for something. (CBQ)

55. Does not become tearful when told to do something s/he does not want to do. (CBQ)

56. Often laughs out loud during play with other children. (CBQ)

57. Prefers quiet activities to active games. (CBQ)

58. Is not shy around new people. (CBQ)

59. Has no trouble sitting still when s/he is told to. (CBQ)

60. Sometimes smiles or giggles when playing by her/himself. (CBQ)

61. Is able to resist laughing or smiling when it is not appropriate. (CBQ)

62. Is comfortable asking other children to play. (CBQ)

63. When drawing or coloring in a book, shows strong concentration. (CBQ)

64. Plays games slowly and deliberately. (CBQ)

65. Does not appear downcast for no reason. (CBQ)

66. Does not become easily frustrated when tired. (CBQ)

67. Talks easily to new people. (CBQ)

68. Is usually pretty excited before leaving on an outing. (CBQ)

69. Often giggles and acts silly. (CBQ)

70. Is good at following instructions. (CBQ)

71. When building or putting something together, becomes very involved in what s/he is doing, and works for long periods. (CBQ)

72. Approaches places s/he has been told are dangerous slowly and cautiously. (CBQ)

73. Gets very enthusiastic about the things s/he does. (CBQ)

74. Rarely becomes discouraged when s/he has trouble making something work. (CBQ)

75. Smiles a lot at people s/he likes. (CBQ)

76. Rarely protests when another child takes his/her toy away. (CBQ)

77. Has difficulty leaving a project s/he has begun. (CBQ)

78. Often laughs out loud in play with other children. (CBQ)

79. Can easily stop an activity when s/he is told "no." (CBQ)

80. Is not easily distracted when listening to a story. (CBQ)

81. Does not get easily irritated when s/he has trouble with some task. (CBQ)

82. Smiles at friendly strangers. (CBQ)

83. Does not get angry when called in from play before s/he is ready to quit. (CBQ)

84. Is usually able to resist temptation when told s/he is not supposed to do something. (CBQ)

85. Sometimes becomes absorbed in a picture book and looks at it for a long time. (CBQ)

86. Likes to sit quietly and watch people do things. (CBQ)

87. Will not get mad when provoked by other children. (CBQ)

88. Smiles when looking at a picture book. (CBQ)

89. Does not have a hard time concentrating on an activity when there are distracting noises. (CBQ)

# 90. Often does not seem to hear me when s/he is working on something. (CBQ)
